# High-quality metagenome-assembled genomes from proximal colonic microbiomes of synbiotic-treated korean native black pigs reveal changes in functional capacity

**DOI:** 10.1038/s41598-022-18503-2

**Published:** 2022-09-15

**Authors:** Jaehoon Jung, Andrew W. Bugenyi, Ma-Ro Lee, Yeon-Jae Choi, Ki-Duk Song, Hak-Kyo Lee, Young-Ok Son, Dong-Sun Lee, Sang-Chul Lee, Young-June Son, Jaeyoung Heo

**Affiliations:** 1 Research Institute of Agriculture and Life Sciences, Seoul National University, Seoul, 151-742 Republic of Korea; 2 eGnome, 26 Beobwon-ro, Songpa-gu, Seoul, 05836 Republic of Korea; 3 Department of Agricultural Convergence Technology, Jeonbuk National University, Jeonju, 54896 Republic of Korea; 4 National Agricultural Research Organization, Mbarara, Uganda; 5 Department of Animal Biotechnology, Jeonbuk National University, Jeonju, 54896 Republic of Korea; 6 International Agricultural Development and Cooperation Center, Jeonbuk National University, Jeonju, 54896 Korea; 7 The Animal Molecular Genetics and Breeding Center, Jeonbuk National University, Jeonju, 54896 Republic of Korea; 8 Department of Animal Biotechnology, Faculty of Biotechnology, College of Applied Life Sciences and Interdisciplinary Graduate Program in Advanced Convergence Technology and Science, Jeju National University, Jeju, 63243 Republic of Korea; 9 Faculty of Biotechnology, College of Applied Life Sciences and Interdisciplinary Graduate Program in Advanced Convergence Technology and Science, Jeju National University, Jeju, 63243 Republic of Korea; 10 Jeju Microbiome Research Center, Jeju National University, Jeju, Jeju Special Self-Governing Province 63243 Republic of Korea; 11 Cronex Co., Cheongju, 28174 Republic of Korea

**Keywords:** Computational biology and bioinformatics, Bacterial genetics

## Abstract

Synbiotics are feed supplements with the potential to promote health and productivity in pigs partly, through modulation of the intestinal microbiome. Our study used shotgun sequencing and 16S rRNA gene sequencing techniques to characterize the effect of a synbiotic containing three Lactobacillus species and a fructo-oligosaccharide on the proximal colonic microbiome of 4- to 7-month-old Korean native black gilts. With shotgun sequencing we constructed unique metagenome-assembled genomes of gut microbiota in Native Black Pig for the first time, which we then used for downstream analysis. Results showed that synbiotic treatment did not alter microbial diversity and evenness within the proximal colons, but altered composition of some members of the *Lactobacillaceae*, *Enterococcaceae* and *Streptococcaceae* families. Functional analysis of the shotgun sequence data revealed 8 clusters of orthologous groups (COGs) that were differentially represented in the proximal colonic microbiomes of synbiotic-treated Jeju black pigs relative to controls. In conclusion, our results show that administering this synbiotic causes changes in the functional capacity of the proximal colonic microbiome of the Korean native black pig. This study improves our understanding of the potential impact of synbiotics on the colonic microbiome of Korean native black pigs.

## Introduction

The Jeju Black Pig is a small, black domestic pig breed that is native to the Korean island of Jeju where it has been conserved for generations^1^. The breed is well adapted to foraging in the rough landscape of the island, where it endures windy conditions and an overall harsh climate^2^. Meanwhile the evolutionary pressure in modern commercial pig farming systems on the mainland has led to the dominance of exotic and commercially developed breeds. Modern commercial breeds have been bred for high performance traits such as prolificacy, mothering ability, number of piglets born, weight gain, growth rate, back fat thickness, lean meat, among others. Despite a low genetic potential for weight gain and overall performance in Jeju black pigs, their pork products have a unique taste which makes them highly sought after and relatively pricier on the market^3,4^. In recent years, there have been efforts to transform Jeju pork production from a traditional backyard system to a more productive, industrialized system while continuing to preserve the breed. Innovative management strategies are being employed to optimize the productivity of this native pig breed. Among these strategies is the use of probiotics, prebiotics and synbiotics to promote health and weight gain in the farmed pigs.

Synbiotics are a combination of probiotics and prebiotics that are used as feed additives or supplements in order to grant health benefits to the host^5^. Probiotics have been defined as “live microorganisms that, when administered in adequate amounts, confer a health benefit on the host”^6^. Prebiotics are dietary components, usually plant fiber that are undigestible by the host but selectively support the growth or activity of a limited group of beneficial microbial genera or species in the gut^7^. These synbiotic combinations modify intestinal microbial composition and influence the development and functioning of the gut with putative benefits to health and performance of pigs. Probiotics containing members of the genus Lactobacillus have been used to modulate the intestinal microbial flora and promote gut health, weight gain and overall performance of pigs^8–10^. This genus is a core member of the pig’s intestinal microbial ecosystem^11^. Lactobacilli are of interest because of their inhibitory role on some potentially pathogenic bacteria in the swine gut. They produce lactic acid which has a disruptive effect on the walls of gram-negative bacteria, such as the *E. coli* and salmonella which are key pathogens in domestic pigs^12,13^. This in addition to their production of antimicrobial peptides^14^ and modulation of immune response in pigs^15^ makes lactobacillus-based probiotics potentially good alternatives to antibiotics in pig farming. Besides contributing to the host’s immune response to enteric pathogens, some strains of *Lactobacillus* promote nutrient digestibility by producing dietary enzymes such as phytases and amylases which then enables breakdown of indigestible dietary components^16^. Several studies have shown that combining prebiotics such as fructo-oligosaccharides with probiotics synergistically enhances the benefits of the probiotic^17,18^. These effects are most important in the colonic environment.

As a site with the highest number of microorganisms, the proximal colon plays very important roles not only in the excretion of waste but also in the absorption of water and electrolytes as well as absorption of vitamins produced by its complex and rich microbial flora^19^. A healthy balance of the microbial ecosystem within the colon is central to its role in the swine gut in that colonic mucosa is the main surface where the gut microbiota and the immune system interact^20^. Therefore, manipulation of this environment to promote the health of Native Black pigs is of interest. The potential of probiotics to promote the productivity of these pigs, can be understood further, by studying their effects on the proximal colonic microbial ecosystem. Metataxonomic techniques such as targeted sequencing of the 16S ribosomal RNA gene and metagenomic techniques like, shotgun sequencing, are frequently used to study microbial composition and infer biological functions in such communities^21,22^. The former approach is cheaper since, by targeting one gene (and typically, a fragment of it), the method imposes lower demands on sequencing coverage, compared to the latter, which probes all prevailing genes in an environment. However, techniques that target marker genes, are limited when studying functional profiles of microbial communities^22^. Even though computational techniques can be used to predict functionality from say, 16SrRNA gene-based taxonomies^23^, shotgun sequencing offers better resolution of functional gene composition within microbial environments^22^. Use of shotgun sequencing data is highly dependent on the completeness of the reference databases for the environment in question. To date, however, the microbiome of Jeju black pig has not been sufficiently studied to support a metagenomic assembly approach.

In this study, we used both shotgun and 16S rRNA sequencing techniques to explore, for the first time, the proximal colonic microbiota of Jeju Native Black pigs. We used a bioinformatic approach to construct a custom database for the Jeju Black pig proximal colonic samples to improve taxonomic classification of shotgun sequence data. We then evaluated the effect of a synbiotic formulation containing *Lactobacillus buchneri* NLRI-1201, *L. plantarum* NLRI-101, *L. casei* DK128 and the prebiotic, fructo-oligosaccharide on the microbial composition within the proximal colon.

## Methods

### Animals and sample collection

All animal experiments were conducted in a facility of contract research organization at Cronex Co., Ltd. (Cheongju, Korea), with following the regulations of the Cronex Co., Ltd. Animal Care and Use Committee (CRONEX-IACUC 202002007). Jeju black pigs (M-Pig®) were purchased from Cronex Co., Ltd. and housed in Cronex Animal SPF (Specific Pathogen Free) facility for whole experimental period, where was environmentally controlled with temperature and relative humidity at 22 ± 2 °C and 50 ± 10%, respectively, under a 12-h light–dark cycle. Each cage (4 pigs per cage, 1.0 m^2^ per pig) was equipped with plastic mesh floor, two round feeders, and two nipple drinkers. Samples were collected from 7-month-old female Jeju black pigs following a 3-month-long experimental study. At the start of the study, eight 4-month-old pigs were randomly recruited into 2 groups (n = 4, in each group). Each group was subjected to a separate diet: either a basal diet (Control group) or synbiotics-supplemented diet (Treatment group). The experimental animals had similar mean body weight both at the start (32.8 ± 4.5 and 32.6 ± 2.5 kg, respectively) and at the end of the experiment (60.4 ± 1.3 and 56.5.x ± 6.8 kg, respectively). All animals were monitored daily by caretakers and the investigating team to ensure their health and welfare. During health monitoring, the care takers observed the animals for signs of GIT conditions (reduced appetite, diarrhea, constipation, rectal prolapses), urogenital tract conditions (discoloration in urine, vaginal discharge) as well as respiratory conditions (coughing, sneezing, rhinitis). All animals remained healthy throughout the study.

The formulation of basal diet is presented in Table 1. The treatment group was allowed ad libitum access to a basal diet supplemented with a synbiotic containing *Lactobacillus buchneri* NLRI-1201 (1.2 × 10^8^ colony forming units (cfu)/kg feed), *L. plantarum* NLRI-101 (1.6 × 10^8^ cfu/kg feed), *L. casei* DK128 (1.4 × 10^8^ cfu/kg feed), and fructo-oligosaccharide (5 g/kg feed). The multi-probiotic and fructo-oligosaccharide from *Saccharum barberi* were purchased from SunBio (Gunpo-Si, Gyeonggi-do, South Korea) and Samyang Corporation (Seongnam-Si, South Korea), respectively. At the end of the 3 months (12 weeks), 8 pigs were sacrificed (4 pigs from each group) and their proximal colonic contents collected for metagenomic analysis. All methods were performed in accordance with the relevant guidelines and regulations of the Cronex Co., Ltd. Animal Care and Use Committee. Genomic DNA (gDNA) from the 8 samples was isolated using DNA extraction kit (NucleoSpin DNA Stool Kit, Macherey–Nagel). After DNA extraction, the metagenome was sequenced and analyzed as the flowchart shown in Fig. S1.

**Table 1:** Composition, nutrient and energy contents of basal diet for Jeju native pig.

Nutrient	Basal diet
**Ingredients, g/kg**	
Corn	392.5
Wheat	200.0
Soybean meal	137.3
Wheat bran	104.0
Soybean hull	54.1
Rice bran	40.0
Fiber feed	20.0
Limestone	15.6
Tallow	10.0
Mono-dicalcium phosphate	9.0
NaCl	5.0
Choline chloride	2.2
Acidifier	2.0
Tryptophan	2.0
Lysin	1.8
Toxin-binder	0.5
Vitamin Premix ^a^	2.0
Mineral Premix ^b^	2.0
Total	1000.00
**Analyzed crude nutrient and energy contents**	
Moisture, %	12.98
Crude protein, %	15.65
Crude fat, %	3.58
Crude ash, %	2.83
Crude fiber, %	5.64
Gross energy, Kcal/kg	3836.00

^a^The vitamin premix provided per kg diet: vitamin A, 12,000 IU; vitamin D3, 2,000 IU; vitamin E, 100 IU; vitamin K3, 4.5 mg; vitamin B1, 2 mg; vitamin B2, 7 mg; vitamin B3, 45 mg; pantothenic acid, 30 mg; vitamin B6, 4.5 mg; Biotin, 0.5 mg; Folic acid, 3.5 mg; vitamin B12, 0.03 mg; antioxidant, 6.6 mg.

^b^The mineral premix provided per kg diet: Fe, 150 mg; Zn, 85 mg; Mn, 37 mg; Cu, 11 mg; Co, 2 mg; I, 0.3 mg; Se, 0.15 mg.

### 16S rRNA metagenome sequencing

From the isolated gDNA, the V3-V4 hypervariable region of the 16S rRNA gene was amplified using the universal prokaryotic primers Bakt_341F (5′-CCTACGGGNGGCWGCAG-3′) and Bakt_805R (5′-GACTACHVGGGTATCTAATCC-3′)^24^. Amplicon libraries were prepared, pooled, and loaded onto an Illumina MiSeq flow-cell using a 600-cycle reagent cartridge (v3 reagent kit). Sequencing was done using a 2 × 301 bp paired-end sequencing method.

### Shotgun metagenome sequencing

In this technique, isolated gDNA was sheared using the S220 Focused-ultrasonicator (Covaris, Inc., Adaptive Focused Acoustics, Massachusetts, US). Library preparation was performed using MGIEasy DNA library prep kit (MGI, Shenzhen, China) according to the manufacturer's instructions. Briefly, After Size-selection of fragmented gDNA using AMPure XP magnetic beads, the fragmented gDNA was end-repaired and A-tailed at 37 °C for 30 min, and 65 °C for 15 min. Indexing adapters were ligated to the ends of the DNA fragments at 23 °C for 60 min. After cleanup of adapter-ligated DNA, PCR was performed to enrich those DNA fragments that have adapter molecules. Thermocycler conditions were as follows: 95 °C for 3 min, 7 cycles of 98 °C for 20 s, 60 °C for 15 s, and 72 °C for 30 s, with a final extension at 72 °C for 10 min. The double stranded library is quantified using QauntiFluor ONE dsDNA System (Promega, Madison, WI, USA). The library was then circularized at 37 °C for 30 min, and then digested at 37 °C for 30 min, followed by cleanup of circularization product. To make DNA nanoball (DNB), the library was incubated at 30 °C for 25 min using DNB enzyme. Finally, the libraries were quantified using QauntiFluor ssDNA System (Promega, Madison, WI, USA). Sequencing of the prepared DNB was conducted on the MGISEQ-2000 system (MGI, Shenzhen, China) with 150 bp paired-end reads. All the sequencing process was conducted by LAS, Inc. (Gimpo, South Korea).

### 16S rRNA metagenome analysis

Denoising and merging, the read pairs were processed using DADA2 package (q2-dada2 v2020.8.0)^25^ (with options *–p-trunc-len-f 275 –p-trunc-len-r 225*). Denoised features were classified by Qiime2 q2-feature-classifier plugin^26^ using a naïve Bayes classifier trained on the V3-V4 hypervariable region of 16S rRNA gene sequences in the Silva Database v138^27^. This denoising and taxonomic classification process was done using the Qiime2 (v2020.08) software^28^. Alpha and beta diversity metrics were calculated using normalized/rarefied sub-samples (14,397 features) of the denoised reads. Analysis of alpha and beta diversity was achieved using Qiime2 diversity core-metrics-phylogenetic plugin and results were visualized in R^29^ using the package ggplot2^30^. Following Thorsen and colleagues’^31^ recommendations for small datasets, we normalized the features using Trimmed Means of M-values (TMM), and explored differentially abundant taxonomy using edgeR^32^.

### De novo assembly and metagenome-assembled genome (MAG) construction

Above all, adapters and low-quality bases (Q < 20) were trimmed using trimmomatic v0.39^33^, and the subsequent reads were mapped to the sus scrofa (Sscrofa11.1) reference genome using a read alignment tool, bowtie2 v2.2.6^34^. Host genome sequences were removed by extracting pair-unmapped reads. To reconstruct metagenome-assembled genomes (MAGs) from our samples, the extracted reads were used as input for de novo assembly using the metaSPAdes v3.14.1 tool^35^.

In order to group the short sequence reads into draft genomes (metagenome binning), reads were first remapped to the assembled contigs with bowtie2^34^, and their depths calculated using the *jgi_summarize_bam_contig_depth* script in MetaBAT2^36^. The metagenome binning process in metaBAT2 tool^36^, used the following options: *–minCV 0.5, –minContig 2,000, –minCVSum 0.5, –maxP 92, –maxEdges 150*. From the constructed bins, low quality bins (< 50% completeness, > 5% contamination) were filtered out using CheckM v1.1.3^37^. Filtered bins were then aggregated and dereplicated using dRep v3.2.0^38^ with a similarity threshold set at 95% (-sa 0.95).

For taxonomic classification of MAGs, the high quality and dereplicated 360 bins were classified by utilizing the GTDB-Tk v1.5.0 software and basing on the Genome Taxonomy Database (GTDB R06-RS202)^39^. The GTDB-Tk tool uses average nucleotide Identity (ANI), phylogenetic tree topology and relative evolutionary divergence (RED) to assign genome-based taxonomies. For downstream analysis in TaxonKit v0.8.0^40^ and kraken2 v2.1.1^41^ the GTDB-based taxonomies were annotated to NCBI taxonomies.

To detect antibiotic-resistance genes within our metagenome samples, we used the ABRicate 1.0.1 tool (https://github.com/tseemann/abricate). We screened our per-sample assembled contigs (metaSPAdes output) based on NCBI AMRFinderPlus database^42^.

### Bioinformatic analysis of read-based taxonomies

A read-based taxonomic classification was conducted using kraken2 v2.1.1 software^41^. We used both the in-built Standard Kraken2 database, and a custom host database to classify the paired end reads from the Jeju Black pig colonic microbial environment. The custom host database was built based on the default database and included the National Center for Biotechnology Information’s (NCBI) RefSeq genomes as well as our classified MAGs. For comparison, reads were aligned to ChocoPhlAn database (2019.01v) using MetaPhlAn3 (v3.0.4), and UniRef90 database using HuManN3 (v3.0.0)^43^.

Following classification in Kraken, and with a normalized read length parameter set at 150, the relative taxonomic abundances of microbes in the samples were re-estimated using bracken v2.6.2 software^44^. Alpha and beta diversity analyses were calculated and visualized using the R package, phyloSeq (v1.34.0)^45^. Differential abundance of the microbes in the samples was statistically analyzed using the edgeR package^32^ in R. Only species with a Log-transformed count-per-million value > 1 and a False Discovery rate (FDR) < 0.01 were considered differentially abundant with statistical significance.

### Gene-based metagenome assembly

For gene-based differential abundance analysis, PLASS assembly (release 4-687d7)^46^ was employed to obtain protein sequences for each sample. Assembled protein sequences were merged, dereplicated and clustered using the *easy-linlinclust* option in MMseqs2 (version 13.45111)^47^ with sequence identity parameter set at 0.5. Sequence searches were then performed using DIAMOND 2.0.11 software^48^ and with the option “*blastx –evalue 0.001 –fast –max-target-seqs 1*”, gene cluster abundances were estimated. Clustered representative sequences were then functionally annotated using emapper-2.1.5^49^ basing on eggnog orthology data^50^. The R package, edgeR^32^ was then used to estimate group specific changes in relative abundances of genes within the colonic microbiomes of the study animals. Statistical significance was considered at FDR < 0.01.

### Ethical approval

All experiments were performed in compliance with the ARRIVE guidelines^51^.

## Results

### 16S rRNA metagenome sequencing

**Figure 1:**
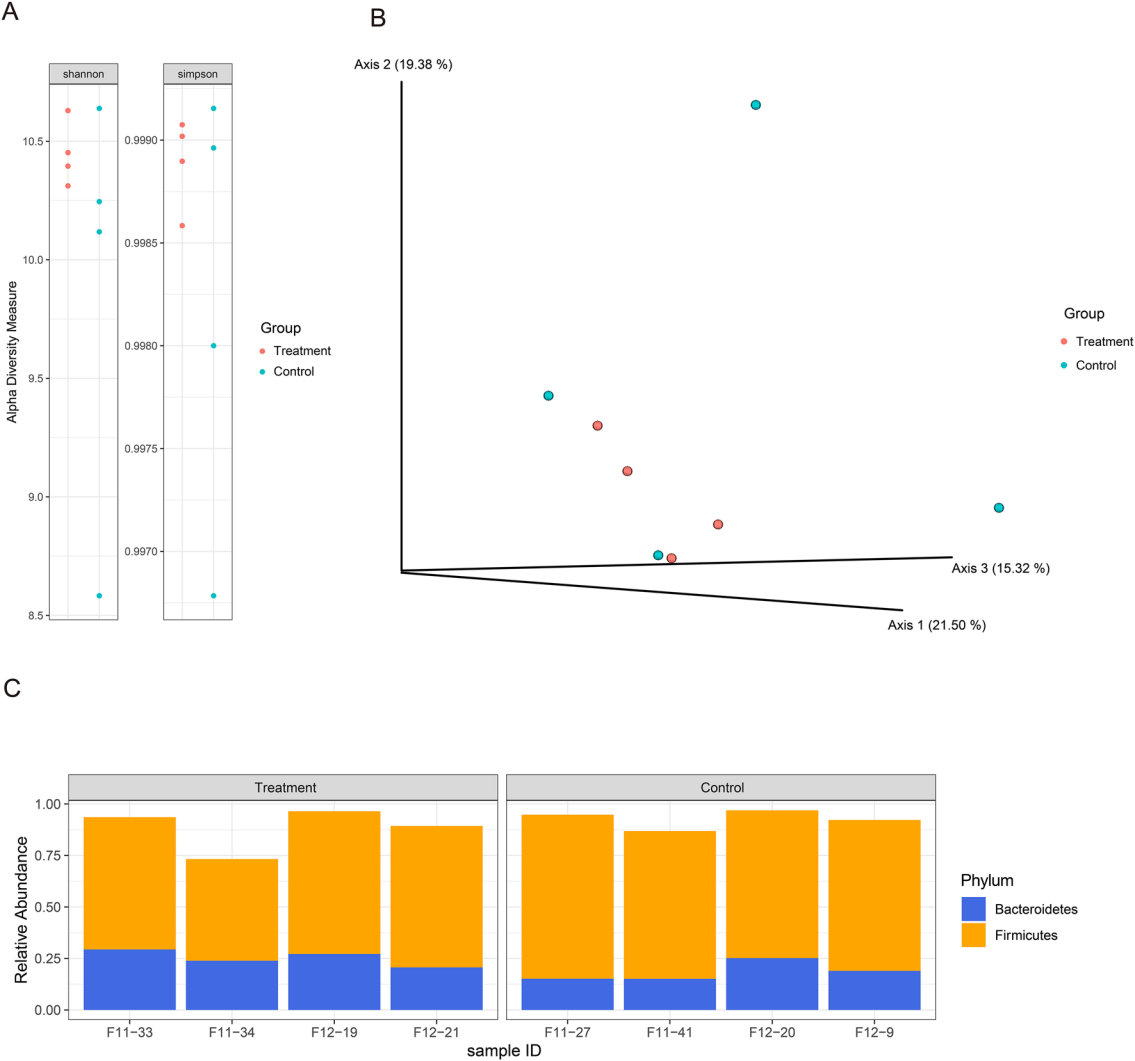
Microbiome profiling results from 16S rRNA gene amplicon sequencing. (**A**) Alpha diversity of the microbial communities within samples in the study groups. (**B**) Principal coordinate analysis (PCoA) plot of the beta diversity based on the Bray–Curtis dissimilarity index between microbial community samples in the study groups. (**C**) Relative composition of the two dominant phyla based on taxonomic analysis of the 16S rRNA amplicon sequence data.

To investigate possible difference in taxonomic composition between the groups, V3-V4 hypervariable regions of the 16S rRNA gene were sequenced from microbial communities in the proximal colon of the study animals. Paired-end sequences were quality-filtered, merged and denoised leaving > 10,000 high quality reads. We assessed possible difference in alpha diversity between the two groups based on 16S rRNA metagenome sequences. Alpha diversity analysis using Shannon and Simpson indices indicated that there was no significance in within-group diversity between our symbiotic-fed animal (treatment group) and controls. The mean number of Shannon index in the treatment group was 10.447 ± 0.135, and 9.896 ± 0.903 for control group. Also, Simpson index was 0.9989 ± 0.0002 and 0.9982 ± 0.0011 for treatment group and control group, respectively (Fig. 1A). Analysis of beta diversity distances using Bray–Curtis dissimilarity showed that symbiotic-fed group is well clustered while control group showed sparse distribution (Fig. 1B).

Taxonomic analysis indicated that *Firmicutes* and *Bacteroidetes* were the most abundant phyla found in the samples. On average, *firmicutes* had a relatively lower abundance in the treatment group (62.8% ± 9.288) compared to the control group (74.1% ± 3.764). Meanwhile, members of the phylum, *Bacteroidetes* had a higher relative abundance (25.3% ± 3.815) in the treatment group compared to controls (18.6% ± 4.728) (Fig. 1C). However, differential abundance in genus level testing between groups found no statistical significance (FDR p-value < 0.05) in the observed differences.

### Shotgun metagenome sequencing

**Figure 2:**
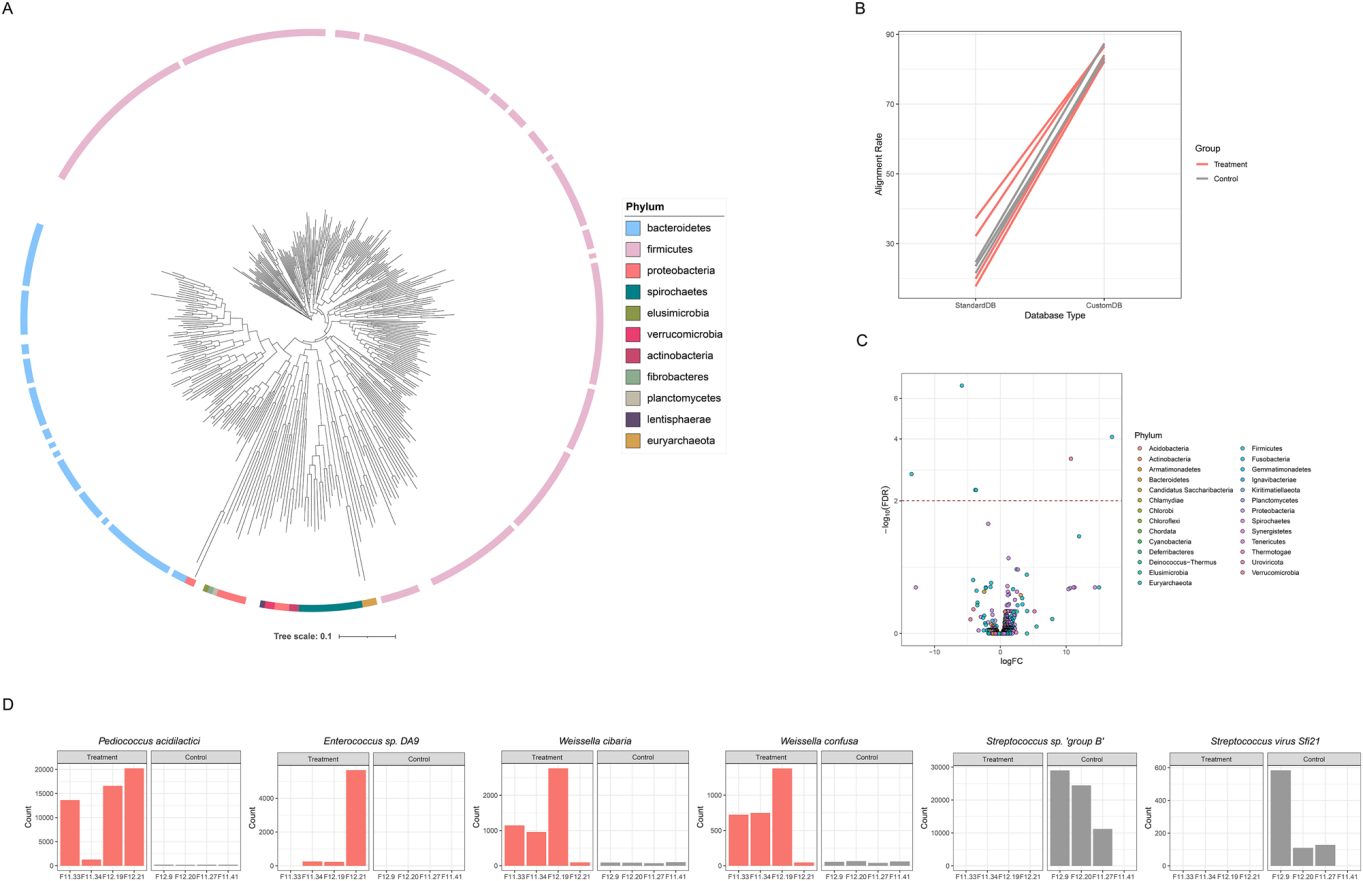
Analysis of the Metagenome-Assembled Genomes (MAGs) obtained using a host-customized database. (**A**) Phylogenetic tree of MAGs (**B**) rate of classified reads using standard database and custom database in kraken2. (**C**) Volcano plot for differentially abundant taxonomies. Each point represents the species-level taxonomies colored with phylum taxa. The taxa are presented with log_2_ fold change (X-axis) and log_10_(FDR-corrected P-value) (Y-axis). Horizontal line represents P-value cut off (FDR P-value 0.01). (**D**) Differentially abundant taxonomies (FDR < 0.01) between groups. Red bars represent composition in samples from the treatment group while gray bars, composition in samples from the control group.

**Figure 3:**
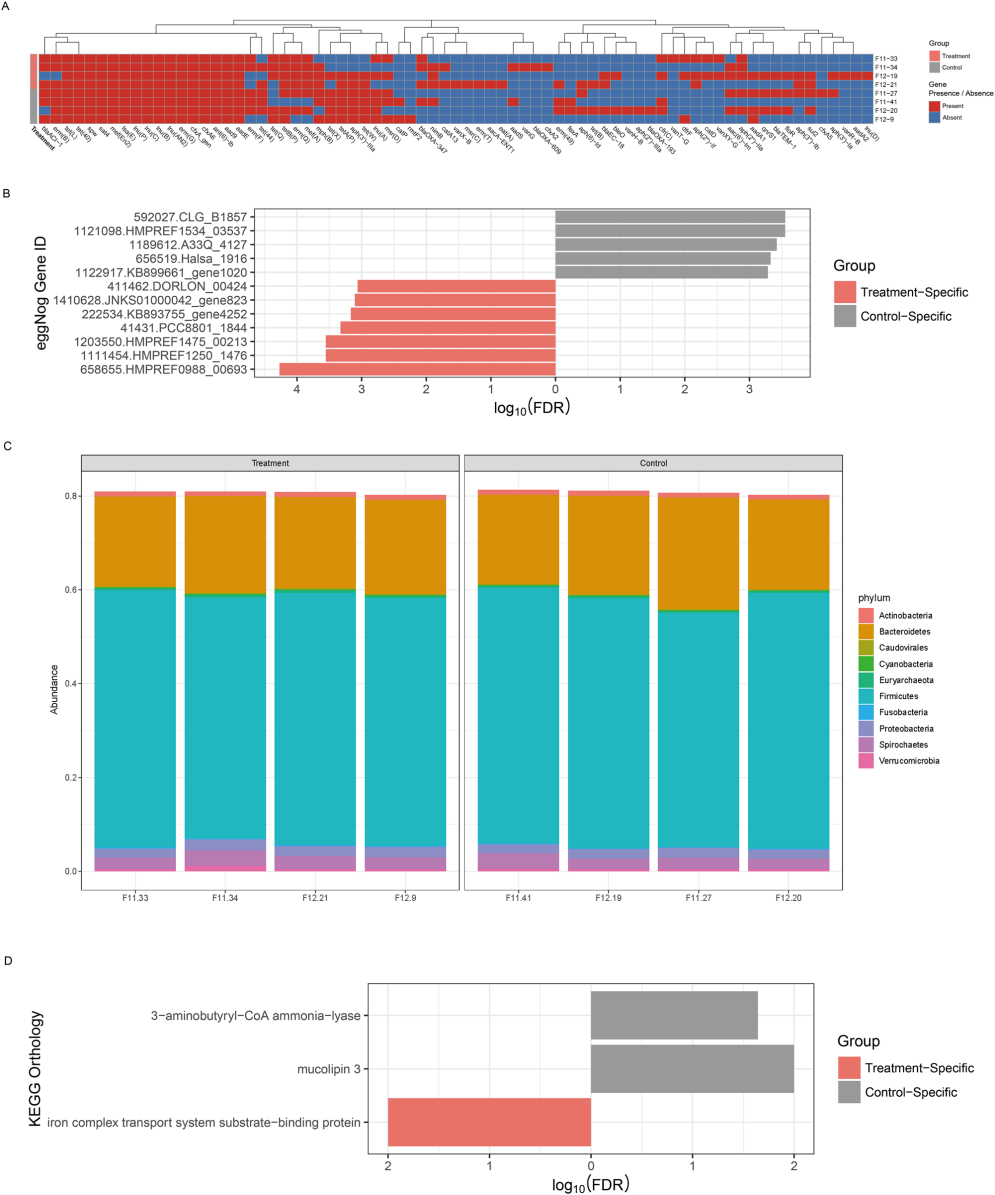
Functional Profiling from shotgun metagenome sequencing. (**A**) Antibiotic resistance genes identified from each sample. (**B**) Bar chart for differentially abundant genes between groups. FDR P-value 0.0005 was used as threshold. (**C**) Taxonomic composition profiling at gene-level. (**D**) Clusters of Orthologous Groups (COG) functional category profiling results. (**E**) Bar chart for differentially abundant KEGG Ortholog functional category. Used FDR P-value was 0.05.

Sequencing on MGISEQ generated 116 Giga Bases with mean quality scores of 34.8 from our samples. After quality trimming and removal of host-derived sequences, about 12.7 Giga Bases (a mean of 43 million read pairs) per sample remained. Single-sample assembly and binning of resulting contigs produced 1,603 bins, which were then quality checked in CheckM (Fig. S2). Concatenation, filtration, and dereplication of all the bins resulted in a total of 360 high-quality metagenome-assembled genomes (MAGs). Taxonomies assigned by a GTDB-based classification of the MAGs as well as the phylogenetic relationships between the MAGs are presented in Fig. 2A. The 360 MAGs were dominated by members of the phyla, Firmicutes (214 MAGs or 59.44%) and Bacteroidetes (73 MAGs making up 20.28%) (Figs. 2A and 3C).

Read-based classification of the shotgun sequences based on the Standard Kraken2 Database yielded a low average alignment (25.07%) (Fig. 2B). To improve classification rate, we built a custom host database which put together our 360 MAGs and the Standard Kraken DB. With this, we were able to achieve a higher average read alignment rate (84.49%). Also, Metaphlan3/Humann3 read classification analysis were conducted and compared with Kraken2 results, showing that almost all of reads were not aligned in nucleotide alignment and about 50 ~ 60% of reads were not aligned in translated alignment (Table S1).

With the read-based taxonomies, we estimated the alpha- and beta-diversities of the microbiota within the proximal colons of the Jeju black pig gilts. By estimating colonic microbial diversities using our shotgun dataset, we obtained lower alpha diversities in the treatment group relative to controls (Fig. S3A). This is in contrast to earlier results from diversity analysis using the 16S rRNA dataset which showed the reverse; higher alpha diversities in the treatment group compared to the controls group (Fig. 1A). However, data from both sequencing methods showed larger deviations in the alpha diversity values within the treatment group compared to the control group. Analysis of Beta diversity distances found no group specific clustering (Fig. S3B).

### Differential abundance analysis

Considering the indistinguishability in abundance and diversity between groups at community level, further analysis was conducted to explore differential abundance at the species level (Fig. 2C). In total, 6 species were identified as statistically different between the treatment group and the control group. Among these species, 5 were bacteria belonging to the firmicutes phylum, while the other was a bacteriophage (*Streptococcus virus* sfi21) (Fig. 2D). On close inspection, the taxa that were overrepresented in the treatment group (treatment-specific taxa) belonged to 2 families; *Lactobacillaceae* family (*Weissella cibaria*, *W. confuse* & *Pediococcus acidilactici*) and *Enterococcaceae* family (*Enterococcus *sp. DA9). On the other hand, the control-specific taxa (*Streptococcus *sp. Group B) belonged to the *Streptococcaceae* family. Also, a *Streptococcus* specific bacteriophage, (*Streptococcus virus* sfi21) was detected only in the control group.

### Functional profiling of pig microbiome

To study the antimicrobial resistome of the proximal colonic samples from our Jeju black pig gilts, we scanned for antibiotic resistance genes within the metagenome assembled contigs. From the samples, we found a total of 73 antibiotic resistance genes (Fig. 3A). The distribution of these AMR genes varied among individuals and showed no differential abundances between groups.

We then investigated the functional role and diversity of proteins encoded by microbial genes associated with colonic microbiomes of the pig samples. To get insights into the gene cluster abundances in metagenomes, gene-based assembly was conducted, and 90,555,907 non-redundant coding sequences were generated. We then assigned functional categories to the genes by mapping them to Clusters of Orthologous Groups (COG) categories. Overall, results showed very little variation in the number of COGs between groups, and samples had coterminous COG profiles (Table S2). Results also showed that 7 COG categories of known functions had average abundances above 5.0% within the samples and together accounted for 53.56% of the total gene abundances. These categories included the COG L (replication & repair), COG G (carbohydrate metabolism and transport), COG M (Cell wall/membrane/envelop biogenesis), COG E (amino acid metabolism and transport), COG J (Translation), COG K (Transcription) and COG C (Energy production and conversion). Differential analysis uncovered 12 differentially abundant genes from 8 COG groups that passed the statistical significance cut-off of FDR corrected p-values < 0.0005 (Table 2, Fig. 3B).

**Table 2:** Differentially abundant genes from protein-level assembly.

eggNOG orthology ID	logFC	FDR	Organism	COG	COG description
HMPREF0988_00693	− 7.14133	5.36E−05	*Lachnospiraceae bacterium*	S	Unknown
HMPREF1475_00213	− 6.10137	2.80E−04	*Prevotella oralis*	S	Unknown
HMPREF1250_1476	− 13.3631	2.80E−04	*Megasphaera *sp. BV3C16-1	Q	Secondary metabolites biosynthesis, transport, and catabolism
HMPREF1534_03537	5.414188	2.80E−04	*Bacteroides massiliensis*	S	Unknown
CLG_B1857	7.929562	2.80E−04	*Clostridium botulinum*	H	Coenzyme transport and metabolism
A33Q_4127	7.210967	3.78E−04	*Indibacter alkaliphilus* LW1	K	Transcription
PCC8801_1844	− 12.8701	4.71E−04	*Cyanothece *sp. PCC 8801	T	Signal transduction mechanisms
Halsa_1916	7.255372	4.71E−04	*Halanaerobium hydrogeniformans*	J	Translation, ribosomal structure and biogenesis
KB899661_gene1020	6.949026	5.19E−04	*Paenibacillus daejeonensis*	G	Carbohydrate transport and metabolism
KB893755_gene4252	− 12.4924	6.81E−04	*Frankia *sp.	L	Replication, recombination and repair
JNKS01000042_gene823	− 5.89862	7.85E−04	*Lachnospiraceae bacterium*	S	Unknown
DORLON_00424	− 7.82383	8.65E−04	*Dorea longicatena*	S	Unknown

Gene-based assignment of taxonomies yielded phylum-level profiles presented in (Fig. 3C). The proportion of the phylum, *Bacteroidetes* was 20.37% (± 0.016), and that of *Firmicutes* was 53.18% (± 0.017). These proportions were in agreement with the 16S rRNA dataset. Protein-level profiles were nearly the same between samples.

We also mapped our data to the Kyoto Encyclopedia of Genes and Genomes (KEGG) database to yield KEGG orthologues. Analysis of the KEGG orthologs revealed a statistically significant increase in the ‘Iron complex transport system substrate-binding protein’ within the treatment group. While the relative increase of the ‘mucolipin 3’ and ‘3-aminobutyryl-CoA ammonia-lyase’ groups in the control group was statistically significant. We however, found no statistically significant differences based on other functional categories (CAZyme, KEGG pathway and PFAM).

## Discussion

In this study, we looked to improve our understanding of the effect of a synbiotic on the colonic microbiome of Korean native black pigs that are traditionally reared on the Jeju Island. We combined shotgun metagenomic sequencing and 16s rRNA marker gene sequencing techniques to study the impact on the microbial composition in the proximal colon of these pigs. The results indicate that the synbiotic causes a subtle shift in the functional profile of the proximal colonic microbiome but no detectable shift in phylogenetic diversity. This is also the first study into the colonic microbiome of this native breed of pigs that are raised under the unique environmental conditions of Jeju Island.

During analysis of the shotgun metagenome sequence data, we encountered a low rate of alignment in the existing reference databases and tools (Fig. 2B, Table S1). One of the reason for this could be the limitation in coverage of a database that is still under curation, considering the quantity of data required to meticulous record all complete bacterial, archaeal, and viral genomes in all environments^52^. Secondly, we suspect that due to the spatial isolation of this native breed of pigs on Jeju Island, their gut environments might represent unique, previously unstudied microbiomes which are not included in the kraken database. To overcome this limitation, we built a custom database for our data through identifying novel MAGs. Consequently 360 novel and high-quality MAGs were identified from the proximal colonic luminal content samples in this study (Fig. 2A). Classification based on the customized database resulted in high-resolution taxonomic composition, with fewer unclassified reads. Although 10 ~ 20% of the reads remained unclassified, we were able to explain more of the unknown reads from the sequence data. In this way, the analysis managed overcome the limitations imposed by a reference database that could not reflect many of the rarely researched microbiota of Native Black Pig.

We found that dietary supplementation with a synbiotic formula had no statistically significant effect on the microbial evenness, alpha- and beta-diversity in the proximal colon of Korean native pigs. The age of the piglets might be one of the reasons why the synbiotic did not have a detectable impact on the community-level microbial diversity^8^. The developing gut of young piglets is more susceptible to significant shifts and becomes less so as it develops into a richer, more mature and stable environment. Our study animals were weaner-growers or sub-adults which have a relatively stable gut microbial composition compared to younger piglets in the peri-weaning phases of growth. Although there was no obvious clustering according to study group, we found some unique/ differentially abundant taxa between the groups. There was an increased representation of some genera of the *Lactobacillaceae* family (*Weissela *spp., *Pediococcus *spp.,) and *Enterococcaceae* (*Enterococcus *spp.) probably due to the stimulatory effect of the probiotic *Lactobacilli*^53^ or the fructo-oligosaccharides in the synbiotic supplement^54^. There was also a reduction in some species of the *Streptococcaceae* family (*Streptococcus *spp.) as well as a *streptococcus* targeting bacteriophage (*Streptococcus virus sfi21*).

In the functional profiling of the proximal colonic microbiomes, we used both shotgun sequence and 16S rRNA marker gene sequence data (Fig. 3). We were able to detect some statistically significant effects of the synbiotic treatment on the distribution of microbial functional gene groups using shotgun sequence data but not with the 16S rRNA sequence data. This was not surprising, as it has been reported in several previous studies that the shotgun sequence data yields more genes and functional metagenomes than the 16S rRNA sequence data^55,56^. During functional profiling, the shotgun sequence data enabled identification of antimicrobial resistance (AMR) genes in the proximal colonic microbiomes of our study animals. We found a total of 73 AMR genes in the proximal colonic microbiome of the study animals, and their abundance did not exhibit group-specific shifts in this study (Fig. 3A). Genes responsible for resistance against tetracyclines, macrolides, aminoglycosides, lincosamides and beta lactams were found in the majority if not all of the samples. This finding in Jeju Black pigs studied here, is similar to findings from several studies that have reported occurrence of resistance genes among commercial pig breeds^57,58^. Pollock and colleagues^57^, found that occurrence and abundance of these AMR genes, however, did not always correspond to antimicrobial exposure. In our study, the animals were not exposed to antibiotics throughout the experimental period, however exposure prior to inclusion into the study was not investigated. Nonetheless, AMR genes are known to be ubiquitous among microbial communities, having coevolved with bacterial strains as a strategy to counter toxic chemicals within their niches^59^.

In addition to identifying antimicrobial resistance genes within this microbial community, the metagenomic analysis provided insights into synbiotic-associated shifts in the functional capacity of the proximal colonic microbiomes (Fig. 3B). The analysis revealed 12 statistically significant differentially distributed gene (belonging to 8 COG groups) between the study groups. Synbiotic treated pigs were enriched in gene categories involved in secondary metabolites biosynthesis, transport, and catabolism (COG Q), Signal transduction mechanisms (COG T) and Replication, recombination, and repair (COG L). The COG Q category includes genes encoding Isochorismatase, an ether hydrolase that catalyzes the synthesis of iron-binding molecules called siderophores. Siderophores have been reported to act as virulence factors^60,61^, and in some instances to mediate microbial interactions that result in suppression of potential gut pathogens^62,63^. Genes in the COG T enable microorganism to sense, and respond to a number of intracellular and extracellular stimuli^64^. Therefore, an abundance of gene families involved in signal transduction could indicate a higher capacity for the bacteria in this group to respond to changes in their environment including nutrient changes, antibiotics, pH among others^65^. While the COG L family of genes is involved in DNA transposition in bacteria. An abundance of this gene family might indicate a high potential for genetic recombination^66^ and/or lateral gene transfer in the microbial community within the proximal colon of synbiotic-fed pigs^67^. Interestingly, some of the relatively increased genes in the treatment group, originated from *Lachnospiraceae bacterium* and *Dorea longicatena*, which are stimulated by fructo-oligosaccharides^68,69^.

We also found that gene families involved in coenzyme transport and metabolism (COG H), transcription (COG K), translation, ribosomal structure, and biogenesis (COG J) as well as carbohydrate transport and metabolism (COG G) were significantly lower in the synbiotic-fed pigs compared to controls. Genes in the COG H category are involved in coenzyme transport and metabolism. Coenzymes are involved in a wide range of intracellular reactions. They play important roles in energy metabolism, cellular response to stress, intracellular redox homeostasis, nucleic acid repair, xenobiotic metabolism, and sometimes, modulation of microbial virulence^70–75^. Genes belonging to the COG K category code for RNA polymerase sigma factors and are involved in initiation of bacterial transcription during exponential growth and gene transcription in response to environmental stressors as well as during morphological changes^76,77^. Fewer COG K genes in the proximal colonic microbiome of synbiotic treated pigs might imply a relative reduction in bacteria with the capacity for rapid multiplication and appropriate response to new environmental stimuli. The COG J category of genes codes for the ribosomal L22 protein which is a structural component of the 50S ribosomal subunit where it binds with multiple domains in the 23S rRNA and facilitates ribosomal assembly^78^. The L22 protein forms part of the peptide exit tunnel of bacterial ribosomes and is therefore thought to regulate translation^79^. The protein also interacts with and mediates susceptibility to macrolides which work by inhibiting translation^80^. COG G is a family of genes that encodes extracellular solute-binding proteins which facilitate bacterial uptake and utilization of carbohydrates^81,82^. Their lower representation could mean a reduced ability to utilize carbohydrates in their environment including the prebiotic studied here^83^. It is worth noting that the some of the significantly increased genes within the control groups, originated from alkaliphilic bacteria (*Indibacter alkaliphilus*, *Halanaerobium hydrogenifromans*, *Paenibacillus daejeonensis*). A possible reason for this might be that relative increase of these genes in the control group microbiomes could be a result of an actual reduction of these genes in the microbiomes of the treatment group and not necessarily an increase in the controls. This is due to the acidic conditions created by the lactic acid producing bacteria in the synbiotic and the relative proliferation of lactobacillus producing bacteria that is stimulated in the treatment group. Overall, the mechanistic association between the synbiotic and the significant differentially expressed genes between the groups is unclear. However, the pattern of differential distribution suggests a shift of functional capacity away from stress tolerance within the synbiotic fed group. This reduced capacity/need for stress tolerance, in turn, could indicate a stabilizing effect of the synbiont on the environment conditions within the proximal colons of the treated pigs.

A major limitation of this study was the small sample size within study groups. Nonetheless, we hope that when interpreted within these limits, the study provides insights into this previously unexplored microbial environment of an unstudied Korean native breed of pigs.

## Conclusion

In this study, we used metagenomic techniques to study the impact of a synbiotic on the proximal colonic microbiomes of Korean native, Jeju black pig gilts. Our results show that the synbiotic, containing *Lactobacillus buchneri* NLRI-1201, *L. plantarum* NLRI-101, *L. casei* DK128 & fructo-oligosaccharide does not cause significant shifts in the microbial abundances, evenness, and diversity at community level. However, the synbiotic increases abundance of members of the family, Lactobacillaceae which have been found to offer health promoting effects to their hosts. Further functional analysis uncovered an effect of the synbiotic on genes belonging to 12 COGs within the colonic microbiome of the Jeju black pigs implying changes in functional capacity of the colonic microbiome. This work improves our understanding of the effect of synbiotics on the microbiome of rare pig breeds such as the Korean black pigs that are native to Jeju Island. We can also infer an understanding of the synbiotics’ potential to promote productivity and industrialization of Korean black pigs.

## Supplementary Information

Supplementary Information.

## Data Availability

Samples are available from Sequence Read Archive (SRA) with the Bioproject accession number PRJNA815969 (https://www.ncbi.nlm.nih.gov/bioproject/PRJNA815969).

## Abbreviations

cfu: Colony forming units
PCR: Polymerase Chain Reaction
rRNA: Ribosomal RNA (ribose nucleic acids)
DNA: Deoxyribose nucleic acids
NCBI: National Center for Biotechnology Information
KEGG: Kyoto Encyclopedia of Genes and Genomes
MAG: Metagenome-assembled genomes
AMR: Antimicrobial resistance
COG: Cluster of Orthologous Groups
