# Supplementary material for: High-quality metagenome-assembled genomes from proximal colonic microbiomes of synbiotic-treated korean native black pigs reveal changes in functional capacity
Source: Sci Rep. 2022 Sep 15;12:14595. doi: 10.1038/s41598-022-18503-2 (PMC9478101; doi:10.1038/s41598-022-18503-2)
Supplement: Supplementary file 1 — Supplementary Information. [file 41598_2022_18503_MOESM1_ESM.docx]

**Supplementary Information for**

**High-Quality Metagenome-Assembled Genomes from Proximal Colonic Microbiomes of Synbiotic-Treated Korean Native Black Pigs Reveal Changes in Functional Capacity**

Jaehoon Jung^1,2^, Andrew W. Bugenyi^3,4^, Ma-Ro Lee^5^, Yeon-Jae Choi^6^, Ki-Duk Song^7^, Hak-Kyo Lee^5,6,7^, Young-Ok Son^8,10^, Dong-Sun Lee^9,10^, Sang-Chul Lee^11^, Young-June Son^11^ & Jaeyoung Heo^5^*

^1^Research Institute of Agriculture and Life Sciences, Seoul National University, Seoul 151-742, Republic of Korea

^2^eGnome, 26 Beobwon-ro, Songpa-gu, Seoul 05836, Republic of Korea

^3^Department of Agricultural Convergence Technology, Jeonbuk National University, Jeonju, 54896, Republic of Korea

^4^National Agricultural Research Organization, Mbarara, Uganda

^5^Department of Animal Biotechnology, Jeonbuk National University, Jeonju, 54896, Republic of Korea

^6^International Agricultural Development and Cooperation Center, Jeonbuk National University, Jeonju, 54896, Korea

^7^The Animal Molecular Genetics and Breeding Center, Jeonbuk National University, Jeonju, 54896, Republic of Korea

^8^Department of Animal Biotechnology, Faculty of Biotechnology, College of Applied Life Sciences and Interdisciplinary Graduate Program in Advanced Convergence Technology and Science, Jeju National University, Jeju, 63243, Republic of Korea

^9^Faculty of Biotechnology, College of Applied Life Sciences and Interdisciplinary Graduate Program in Advanced Convergence Technology and Science, Jeju National University, Jeju, 63243, Republic of Korea

^10^Jeju Microbiome Research Center, Jeju National University, Jeju Special Self-Governing Province, 63243, Republic of Korea

^11^Cronex Co., Cheongju, 28174, Republic of Korea

***Correspondence:** jyheo@jbnu.ac.kr (J.H.); Tel.: +82-63-270-2549 (J.H.)

**Table of contents**

**Supplementary Figures**

**Figure S1** | Flowchart for the analysis from sequencing to functional annotation

**Figure S2** | CheckM completeness estimates for 1,603 assembled bins from gut metagenomes

**Figure S3** | Microbiome diversity analyses using shotgun metagenome sequencing

**Supplementary Tables**

**Table S1** | Unaligned rate comparison between Metaphlan3/Humann3 and Kraken analysis

**Table S2** | Relative abundance of COG categories among the samples

**Supplementary Figures**


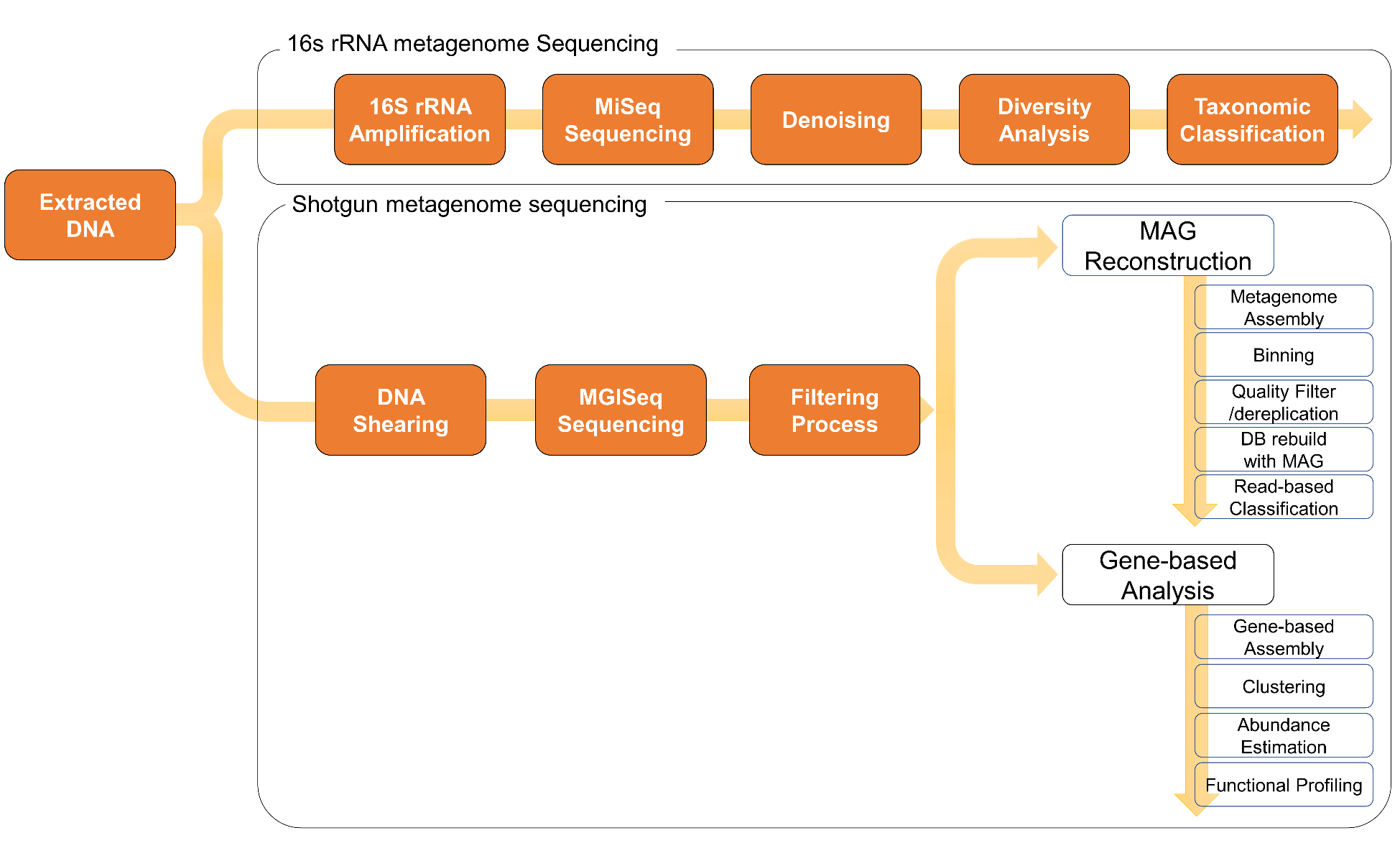


**Figure S1.** Flowchart for the analysis from sequencing to functional annotation.


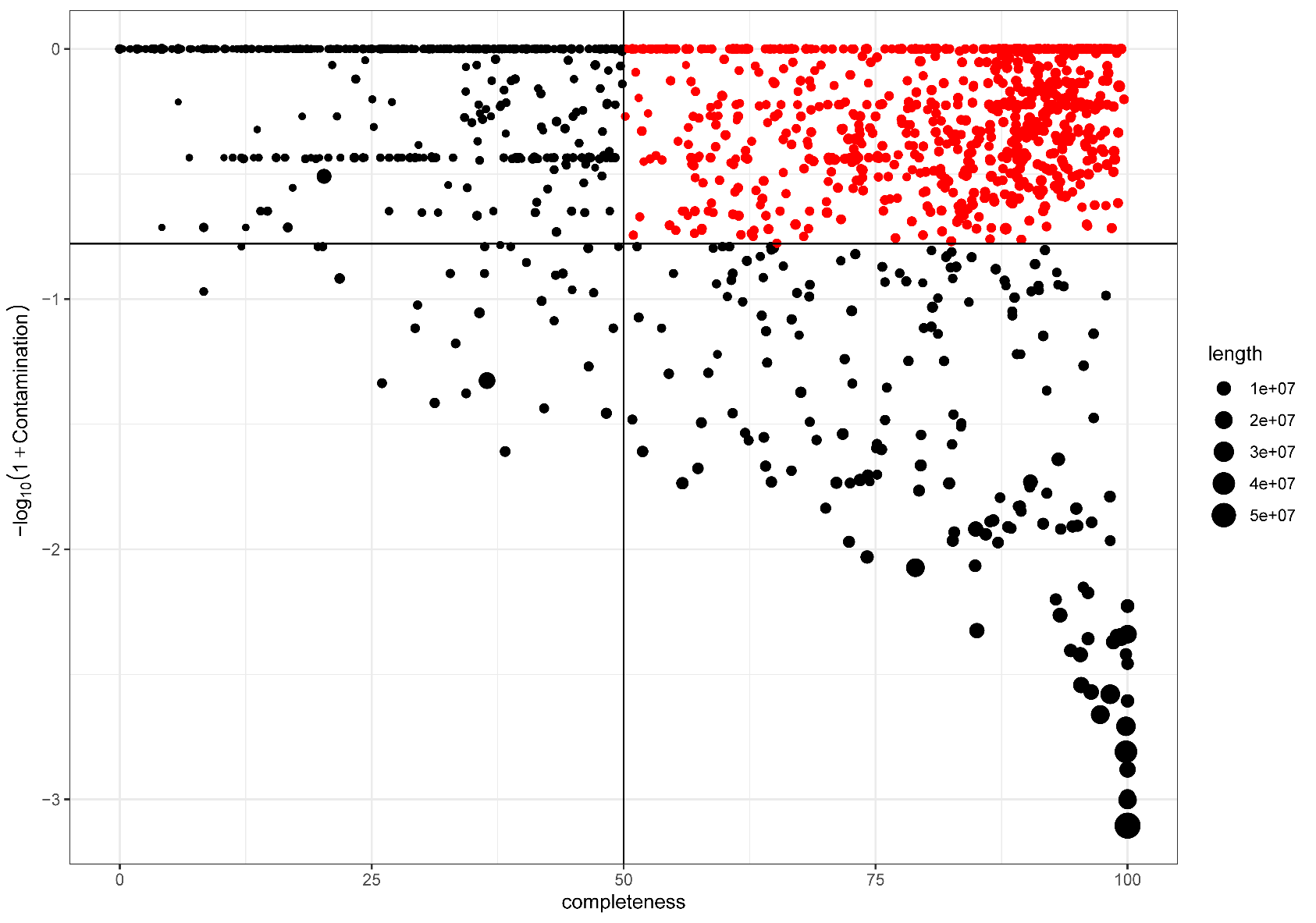


**Figure S2. CheckM completeness estimates for 1,603 assembled bins from gut metagenomes**. The size of the point on the scatter plot corresponds to the bin length, and dots were represented as completeness (X-axis) and –log_10_(1+contamination) (Y-axis). Red dots represent 360 bins which passed the cut-off criteria (completeness ≥ 50, contamination ≤ 5).


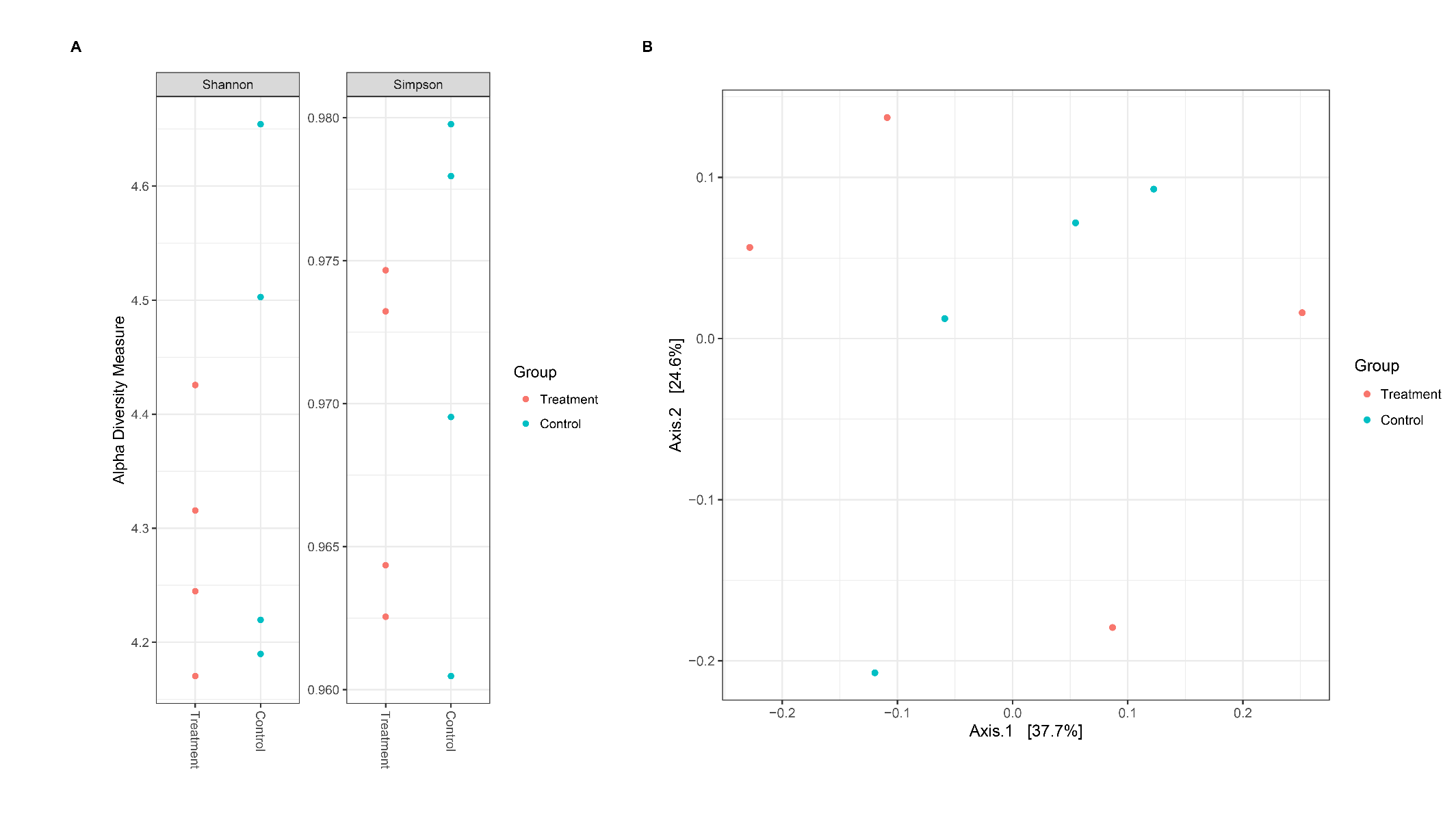


**Figure S3. Microbiome diversity analyses using shotgun metagenome sequencing**. **A.** alpha diversity analysis within samples from the treatment group and the control groups. **B.** A 2-dimensional PCoA plot based on the Bray-Curtis distances showing the distribution of samples from the Treatment and control groups.

**Supplementary Tables**

**Table S1.** Unaligned rate comparison between Metaphlan3/Humann3 and Kraken analysis

| **Sample Name** | **Group** | **Unaligned reads ratio of MetaPhlAn3/HuMAnN3** | | **Unaligned reads ratio of Kraken** | |
| --- | --- | --- | --- | --- | --- |
|  |  | **MetaPhlAn3 Nucleotide Alignment** | **HuMAnN3 Translated Alignment** | **Standard DB** | **Custom DB** |
| F11-33 | Treatment | 91.26% | 59.20% | 67.84% | 12.95% |
| F11-34 |  | 97.05% | 68.08% | 82.22% | 17.59% |
| F12-19 |  | 86.61% | 54.33% | 62.72% | 13.51% |
| F12-21 |  | 96.65% | 66.50% | 80.05% | 16.57% |
| F11-27 | Control | 91.47% | 57.78% | 76.46% | 17.90% |
| F11-41 |  | 95.22% | 65.38% | 78.45% | 15.95% |
| F12-9 |  | 93.94% | 61.31% | 76.39% | 17.00% |
| F12-20 |  | 90.25% | 54.61% | 75.30% | 12.60% |

**Table S2.** Relative abundance of COG categories among the samples

| COG | COG description | Relative abundance | | | | | | | | Average abundance |
| --- | --- | --- | --- | --- | --- | --- | --- | --- | --- | --- |
|  |  | **F11-33** | **F11-34** | **F12-21** | **F12-9** | **F11-41** | **F12-19** | **F11-27** | **F12-20** |  |
| S | Function Unknown | 16.036 | 15.940 | 15.883 | 15.981 | 15.986 | 16.094 | 16.176 | 16.046 | 16.0176 |
| L | Replication, recombination, and repair | 12.879 | 12.679 | 12.644 | 12.820 | 12.753 | 12.883 | 12.573 | 12.783 | 12.7518 |
| G | Carbohydrate metabolism and transport | 8.523 | 8.594 | 8.760 | 8.577 | 8.653 | 8.517 | 8.631 | 8.476 | 8.5915 |
| M | Cell wall/ membrane/ envelope biogenesis | 7.900 | 8.031 | 7.994 | 7.923 | 7.943 | 7.920 | 8.057 | 7.885 | 7.9566 |
| E | Amino Acid metabolism and transport | 7.090 | 6.966 | 7.034 | 7.094 | 6.994 | 7.040 | 7.043 | 7.032 | 7.0365 |
| J | Translation, ribosomal structure & biogenesis | 6.243 | 6.183 | 6.204 | 6.311 | 6.182 | 6.253 | 6.186 | 6.269 | 6.2289 |
| K | Transcription | 5.691 | 5.598 | 5.628 | 5.570 | 5.746 | 5.585 | 5.498 | 5.766 | 5.6352 |
| C | Energy production and conversion | 5.383 | 5.288 | 5.353 | 5.332 | 5.315 | 5.353 | 5.391 | 5.478 | 5.3616 |
| P | Inorganic ion transport and metabolism | 4.884 | 4.854 | 4.882 | 4.935 | 4.862 | 4.999 | 5.015 | 4.859 | 4.9112 |
| T | Signal transduction mechanisms | 3.798 | 4.053 | 3.952 | 3.790 | 3.979 | 3.778 | 3.791 | 3.874 | 3.8770 |
| H | Coenzyme transport and metabolism | 3.634 | 3.731 | 3.647 | 3.652 | 3.639 | 3.660 | 3.786 | 3.616 | 3.6707 |
| F | Nucleotide metabolism and transport | 3.478 | 3.402 | 3.424 | 3.499 | 3.404 | 3.489 | 3.456 | 3.401 | 3.4443 |
| O | Post-translational modification, protein turnover, chaperone functions | 3.194 | 3.212 | 3.179 | 3.237 | 3.160 | 3.208 | 3.199 | 3.229 | 3.2021 |
| V | Defense mechanisms | 2.825 | 2.832 | 2.846 | 2.809 | 2.847 | 2.799 | 2.737 | 2.787 | 2.8102 |
| U | Intracellular trafficking, secretion, & vesicular transport | 2.343 | 2.394 | 2.383 | 2.346 | 2.359 | 2.348 | 2.391 | 2.358 | 2.3652 |
| I | Lipid transport and metabolism | 2.013 | 2.042 | 2.028 | 2.029 | 2.032 | 2.014 | 2.021 | 2.014 | 2.0240 |
| D | Cell cycle control, cell division, chromosome partitioning | 1.974 | 1.960 | 1.956 | 1.984 | 1.971 | 1.966 | 1.964 | 2.013 | 1.9734 |
| N | Cell motility | 1.154 | 1.291 | 1.231 | 1.166 | 1.209 | 1.154 | 1.165 | 1.139 | 1.1886 |
| Q | Secondary metabolites biosynthesis, transport, and catabolism | 0.805 | 0.797 | 0.814 | 0.782 | 0.810 | 0.777 | 0.770 | 0.813 | 0.7960 |
| Z | Cytoskeleton | 0.084 | 0.086 | 0.082 | 0.087 | 0.088 | 0.084 | 0.078 | 0.088 | 0.0845 |
| A | RNA processing and modification | 0.036 | 0.036 | 0.038 | 0.039 | 0.034 | 0.038 | 0.039 | 0.035 | 0.0368 |
| W | Extracellular structures | 0.018 | 0.017 | 0.018 | 0.018 | 0.018 | 0.023 | 0.017 | 0.019 | 0.0184 |
| B | Chromatin Structure and dynamics | 0.017 | 0.016 | 0.017 | 0.018 | 0.017 | 0.018 | 0.016 | 0.018 | 0.0170 |
| Y | Nuclear structure | 0.001 | 0.001 | 0.001 | 0.001 | 0.001 | 0.001 | 0.001 | 0.001 | 0.0008 |
